# Bacteriophages as Biocontrol Agents for *Flavobacterium psychrophilum* Biofilms and Rainbow Trout Infections

**DOI:** 10.1089/phage.2020.0021

**Published:** 2020-12-16

**Authors:** Krister Sundell, Lotta Landor, Daniel Castillo, Mathias Middelboe, Tom Wiklund

**Affiliations:** ^1^ Laboratory of Aquatic Pathobiology, Environmental and Marine Biology, Åbo Akademi University, Turku, Finland.; ^2^ Marine Biological Section, Department of Biology, University of Copenhagen, Helsingør, Denmark.

**Keywords:** bacteriophage, *Flavobacterium psychrophilum*, phage:host ratio, PHR, biofilm, rainbow trout, *Oncorhynchus mykiss*

## Abstract

***Background:*** Bacteriophages (phages) have been proposed as an alternative to antibiotics and surface disinfectants for treatment of *Flavobacterium psychrophilum* biofilms and fish infections in aquaculture settings. The aim of the study was to estimate the minimal phage:host ratio (PHR) required for the control of *in vitro* biofilm formation and mortalities caused by *F. psychrophilum* in experimentally infected fish.

***Materials and Methods:*** *F. psychrophilum* cells in different stages of biofilm formation were exposed to the lytic phage FPSV-D22 at different PHRs.

***Results:*** Our results show that an initial PHR of 0.01 is sufficient for more than an 80% inhibition of attachment and colonization, and disruption of maturated *F. psychrophilum* biofilms, whereas greater ratios resulted in almost complete interruption of the different biofilm stages. Interestingly, a similar response was observed in a phage therapy trial with live rainbow trout (*Oncorhynchus mykiss*), where treatment of *F. psychrophilum*-infected fish by injection of serial bacteriophage doses resulted in significantly (***p ≤ 0.001) higher survival already at a PHR of 0.02.

***Conclusions:*** These results indicate that phages have the potential to be effective for control and treatment of *F. psychrophilum* infections in fish farms even when applied in concentrations lower than previously expected.

## Introduction

*F**lavobacterium psychrophilum* is a Gram-negative bacterium and the etiological agent of bacterial cold-water disease (BCWD) causing mortalities in several farmed salmonid species and substantial economic losses for the fish farming industry. As efficient commercially available universal vaccines are still not available despite several decades of effort in vaccine development, the use of antibiotics such as florfenicol and oxytetracycline remains the main choice for treatment of BCWD outbreaks today.^1^ The increasing problems with antibiotic resistance have ignited research interest into the potential use of bacteriophages (phages) as targeted biological weapons against specific pathogenic bacteria in the food production industry, including the aquaculture sector.

*F. psychrophilum* biofilms attached to living or inert surfaces in aquaculture settings have the potential to withstand antibiotic treatment and may constitute a reservoir for strains causing recurring infections in farmed fish.^2,3^ Biofilm disinfection in aquaculture is, however, a laborious process, including the use of environmentally toxic chemicals that could both cause harm for living organisms and corrode or damage aquaculture equipment. *F. psychrophilum* biofilms can potentially preserve virulence factors and, if exposed to sublethal antimicrobial concentrations, develop tolerance to antimicrobial compounds.^3,4^ The use of phages as disinfectants of *F. psychrophilum* biofilms has been suggested previously, as certain phages and phage combinations showed inhibiting and biomass reducing properties.^5^

Studies with experimentally infected salmonids have shown that *F. psychrophilum*-specific phages, when administered by intraperitoneal (i.p.) injection, are able to maintain their bactericidal activity over time in the inner organs of fish and significantly reduce BCWD mortalities when applied in a 10:1 phage:host ratio (PHR).^6,7^ For the treatment and prevention of biofilms and *F. psychrophilum* infections in aquaculture settings, high local phage concentrations corresponding to a PHR of 10 could, however, be difficult to achieve.

The aim of this study was to investigate the antibiofilm potential of lytic bacteriophages against *F. psychrophilum* and their efficacy against rainbow trout infection at different initial PHRs. Therefore, we examined the minimal phage dosage required to inhibit different stages of *F. psychrophilum* biofilm formation *in vitro* and to reduce BCWD mortalities in fish.

## Materials and Methods

### Bacteriophages

The lytic bacteriophages used in this study were the previously isolated FPSV-D22 (*Siphoviridae*; NCBI:txid2575341) and FpV4 (*Podoviridae*; NCBI:txid1740108), which both have shown a broad host-range against virulent *F. psychrophilum* strains.^8–10^ For the biofilm experiments, the FPSV-D22 filtrate (∼10^11^ plaque forming units [PFU]/mL) was purified by diafiltration and suspended in tris-magnesium (TM) buffer (50 mM Tris HCl, 10 mM MgSO_4_ at pH 7.5) and stored at 4°C in the dark. For the fish experiments, 1 L of bacteria cultures grown to an optical density (OD) of 0.2 at 600 nm were infected with the phages FPSV-D22 and FpV4 at a PHR of 1. The lysed bacterial cultures were centrifuged (9000 *g*, 10 min, 4°C) and filtered through a 0.2 μm-pore size sterile filter. Then, the phage stocks were concentrated by adding polyethylene glycol 8000 and NaCl (final concentration 10% w/v and 5.8%, respectively) before incubation at 4°C for 24 h. Subsequently, phage solutions were centrifuged (10,000 *g*, 30 min, 4°C) and the phage pellet was resuspended in 200 mL of saline magnesium buffer (50 mM Tris-HCl, pH 7.5, 99 mM NaCl, 8 mM MgSO_4_). A combination (1:1, v/v) of the bacteriophage suspensions consisting of FPSV-D22 (2.2 × 10^9^ PFU/mL) and FpV4 (1.2 × 10^9^ PFU/mL) was used both undiluted (1:1) and in dilutions 1:100 and 1:10,000 in TM buffer for injection into the fish.

The concentration of phages used in the different experiments was verified using the double-layer agar plaque assay where an overnight tryptone yeast extract salts (TYES) broth culture (OD_520_ = 0.2 ± 0.05) of the proliferation host *F. psychrophilum* FPS-S6 was used to produce a bacteria-infused top agar by mixing 300 μL of the FPS-S6 broth culture with 4000 μL of 45°C melted TYES agar (0.4% agar).^9^ The bacteria–agar mixture was briefly mixed by vortexing and poured over a dry underlay TYES agar plate. The solidified plates were kept at 4°C and used within 3 h. Phage dilutions used in biofilm experiments were pipetted in 5 μL drops onto the double-layer agar plates and incubated at 15°C for 3–5 days before plaque counting.

### *F. psychrophilum* isolates

Four *F. psychrophilum* isolates, FPS-S6, 160401-1/5N, FPS-R9, and 950106-1/1, were selected for this study based on their previously determined virulence and capacity to adhere to polystyrene.^8^ Isolates FPS-S6, 160401-1/5N, and FPS-R9 were used for the biofilm experiments, whereas isolate 950106-1/1, a model strain for fish challenge studies and phage–host interactions, was used for the fish experiment.^6–10^ All isolates were derived from infected rainbow trout and showed a smooth colony phenotype when grown on TYES agar.^11^ In our previous study, three of the isolates (FPS-S6, 160401-1/5N, and 950106-1/1) were susceptible to both FPSV-D22 and FpV4 phages *in vitro*, whereas one of the isolates (FPS-R9) was found to be intrinsically resistant to the phages in question and was included in the biofilm testing as a negative control of the methodology.^8^

### Preparation of bacterial test suspensions

For the biofilm studies, bacterial suspensions of each isolate were produced by scraping of *F. psychrophilum* cells from 3-day-old TYES agar cultures and subsequent inoculation in sterile TYES broth.^11^ The OD of the suspensions were adjusted to 0.45 ± 0.05 at 520 nm, corresponding to an approximate concentration of 5.0 × 10^8^ colony forming units (CFU)/mL, followed by a 1:100 dilution in filtered (0.22 μm) and autoclaved lake water (FALW) giving the test suspensions a final concentration of 5.0 × 10^6^ CFU/mL.

### Determination of optimal incubation time for biofilm maturation

To estimate the optimal incubation time for *F. psychrophilum* biofilm maturation before the detachment phase, the bacterial biomass of isolate FPS-S6 attached to polystyrene wells (Nunclon™ Delta surface) was quantified using a previously described crystal violet (CV) staining method after 3, 5, and 7 days of incubation at 15°C (Ref.^12^). In brief, 20 μL of the prepared bacterial test suspension (∼10^5^ CFUs) was added to wells containing 180 μL of FALW and incubated for 1 h at 15°C. After the incubation, planktonic cells from the wells were carefully removed by pipetting, and the adhered cells were supplemented with 200 μL of fresh TYES broth for another incubation (3, 5, and 7 days) at 15°C in a humid chamber. After the second incubation, the contents of the plates were discarded, and the plates were washed three times by submersion in a tap water bath and the wells were subsequently air dried. The wells were then stained with 220 μL of 0.1% CV, and incubated at room temperature for 45 min. The stain was removed from the wells and the plates were washed three times by submersion in tap water baths, and air dried. To solubilize the CV, 94% ethanol was added (220 μL) to each well and left to incubate for 15 min. From each well, 100 μL of solubilized CV was transferred into a clean 96-well microtiter plate and the absorbance of each well in the plate was measured at 595 nm using an absorbance microplate reader. Six replicate wells and three replicate plates were used for each incubation time.

### Phage prevention of biofilm attachment

To assess the ability of FPSV-D22 to prevent initial bacterial attachment onto a polystyrene surface and subsequent biofilm formation, phages were added to the wells of a microtiter plate before inoculation of *F. psychrophilum* cells. First, the stock solution of FPSV-D22 was diluted 1:100 in FALW to an approximate concentration of 10^9^ PFU/mL. Then, a 10-fold serial dilution of FPSV-D22 was prepared in FALW before adding 180 μL of the dilutions in a series ranging from 10^7^ to 10^2^ PFU/well in hexaplicate into a flat-bottomed 96-well microtiter plate (Nunclon Delta surface). Wells with phage-free FALW (180 μL) were used as negative controls. Then, 20 μL of the bacterial test suspension (∼10^5^ CFUs) was added to each treatment containing 180 μL of 10-fold serially diluted phage concentrations at an initial PHR ranging from 100 to 0.001. Bacteria-free wells were used as a phage concentration control, to which 20 μL of FALW was added. After incubation for 1 h at 15°C, allowing adhesion of viable cells to the walls, the contents of the wells were carefully removed, and 200 μL of TYES broth was added to each well to allow for growth of attached bacterial cells.^13^ The plate was incubated statically in a humid chamber for 3 days at 15°C before the CV staining and absorbance measuring procedure described earlier. Six replicate wells and three replicate plates were used for each treatment.

### Phage interruption of colonization and biofilm formation

To assess the ability of FPSV-D22 to interrupt colonization of polystyrene surfaces and subsequent biofilm formation, phages were added shortly after inoculation of *F. psychrophilum* cells to a 96-well flat-bottomed microtiter plate (Nunclon Delta surface). To allow bacterial attachment and colonization, 20 μL of the bacterial test suspension (∼10^5^ CFUs) was added to wells containing 180 μL of FALW and incubated for 1 h at 15°C. After the incubation to allow bacterial adhesion, the contents of the wells were carefully removed, and the number of attached bacterial cells were determined in separate control wells by resuspending the cells in a 200 μL volume of TYES broth supplemented with 0.1% saponin before enumeration by dilution and plating onto TYES agar.^3^ Then, a 10-fold serial dilution of phage FPSV-D22 in TYES broth was added (200 μL/well) to the colonized wells corresponding to a PHR ranging from 100 to 0.001. Bacteria-free and phage-free wells to which 200 μL TYES broth were added were used as controls. The microtiter plate was then incubated statically for 3 days at 15°C in a humid chamber before CV staining and absorbance measuring. Six replicate wells and three replicate plates were used for each treatment.

### Phage disruption of maturated biofilms

To assess the ability of FPSV-D22 to disrupt maturated *F. psychrophilum* biofilms, phages were added after allowing for biofilm formation on the polystyrene surfaces. Before addition of phages, 20 μL of the bacterial test suspension (∼10^5^ CFUs) was added to 180 μL of FALW in wells of a 96-well flat-bottomed microtiter plate (Nunclon Delta surface) and bacteria-free wells were used as a control. For bacterial attachment, the plate was incubated for 1 h at 15°C after which the contents of the wells were carefully removed. Then, 200 μL of fresh TYES broth was added to each test well and the plates were placed in a humid chamber and incubated statically for 3 days at 15°C to allow for biofilm maturation. After the 3-day incubation, the contents of the microtiter plate were again carefully removed and to estimate the bacterial concentration of the mature biofilm, attached bacterial cells in separate control wells were enumerated as described earlier. Then, a 10-fold serial dilution series of the phage FPSV-D22 filtrate ranging from 10^7^ to 10^3^ PFU/mL was prepared in TYES broth and added (200 μL/well) to the test wells. Bacteria-free and phage-free test wells were used as controls. The plate was then placed in a humid chamber and incubated statically for 3 days at 15°C before CV staining and absorbance measuring. Six replicate wells and three replicate plates were used for each treatment.

### Estimation of the antibiofilm potential of lytic bacteriophages

The concentrations of phages used in the *in vitro* biofilm experiments were determined at the start of each experiment using the previously described double-layer agar plaque assay.^9^ Bacterial cell concentrations were determined by standard CFU counting to estimate the initial PHR under the experimental conditions used (Tables 1 and 2). The percentage inhibition of different biofilm stages by each tested phage concentration was calculated according to the following: % inhibition = 100 × [1 – (A_X_ – A_Min_)/(A_Max_ – A_Min_)], where A_X_ is the measured absorbance at a given initial PHR, and A_Min_ and A_Max_ the minimum (negative control: only phages) and maximum (positive control: only bacteria) mean absorbance values, respectively, of the test.

**Table 1.** Phage Concentrations

Isolate	Biofilm attachment	Biofilm colonization	Maturated biofilm
FPS-R9	2.4 × 10^7^	1.8 × 10^7^	2.8 × 10^7^
FPS-S6	1.6 × 10^7^	1.4 × 10^7^	1.4 × 10^7^
160401-1/5N	3.6 × 10^7^	2.0 × 10^7^	1.8 × 10^7^

Highest concentration (PFU/mL) of bacteriophage FPSV-D22 calculated for biofilm experiments at different stages, estimated from plaque assays with proliferation host isolate FPS-S6.

PFU, plaque forming units.

**Table 2.** Bacterial Concentrations

Isolate	Biofilm attachment	Biofilm colonization	Maturated biofilm
FPS-R9	4.0 × 10^5^	2.9 × 10^4^	1.1 × 10^8^
FPS-S6	1.2 × 10^4^	2.1 × 10^4^	1.2 × 10^8^
160401-1/5N	4.0 × 10^5^	4.1 × 10^4^	1.3 × 10^8^

Concentrations (CFU/mL) for each *Flavobacterium psychrophilum* isolate under three different stages of the biofilm experiments.

CFU, colony forming units.

### Efficacy of bacteriophages on survival of experimentally infected fish

To estimate the efficacy of phages on survival of experimentally infected fish *in vivo* at different PHRs, 30 infected rainbow trout individuals (∼8 g) were treated with 3 different phage concentrations and divided into 3 identical 150 L tanks with flow through of dechlorinated tap water (∼12°C) and aeration. Equal-sized negative (only phages) and positive (only bacteria) control groups were included in the study. All fish were fed with commercial 1.2 mm fish feed (Rehuraisio) throughout the experiment. Before the challenge, *F. psychrophilum* isolate 950106-1/1 was incubated for 2 days in TYES broth with agitation, washed by centrifugation (5000 *g*, 15 min, 4°C) and resuspended in fresh TYES broth to an OD_520_ = 1, which corresponded to a bacterial concentration of 1.6 × 10^9^ CFU/mL as determined by dilution and colony plate count. Before injection, the bacterial suspension was diluted 1:2 in TYES broth, which has been shown to be nontoxic to rainbow trout.^6^ Fish were anesthetized by immersion in a 0.05 g/L bath solution of benzocaine and i.p. injected with 0.1 mL of the bacterial suspension. One day postchallenge with 8 × 10^7^ CFU of *F. psychrophilum* 950106-1/1, the phage treatment groups of fish were anaesthetized and injected i.p. with a cocktail of the bacteriophages FPSV-D22 and FpV4 in three different calculated PHRs; 2, 0.02, and 0.0002. The mortality after challenge was monitored for 21 days during which dead and moribund fish were removed for bacteriological examination. Isolated yellow colonies were identified as *F. psychrophilum* by PCR using species-specific primers.

After the challenge experiment, the efficacy of each treatment was estimated by calculation of the relative percentage survival (RPS).^14^ For each treatment group, RPS was calculated according to the formula: RPS = 1 – (% mortality in treatment group/% mortality in control group) × 100. Kaplan–Meier survival curves were generated for each treatment group and compared statistically with the positive control group (bacteria only) using the log-rank (Mantel–Cox) test in GraphPad Prism 8.4.2 followed by pairwise comparison using the Gehan–Breslow–Wilcoxon test.

### Ethics statement

The animal experiments were performed in Finland under project (ESAVI/4225/04.10.07/2017) and personal license issued by the National Animal Experimental Board (Eläinkoelautakunta, ELLA).

## Results

### Determination of optimal incubation time for biofilm maturation

Under the present experimental conditions, the highest biofilm formation of isolate FPS-S6 was obtained after 3 days of incubation at 15°C. The mean absorbance measured at 595 nm declined from 0.414 after 3 days of incubation to 0.299 and 0.202, after 5 and 7 days of incubation, respectively. Therefore, the 3-day incubation time was selected for following studies involving biofilm maturation before detachment of cells.

### Estimation of the antibiofilm potential of lytic bacteriophages

**FIG. 1.**
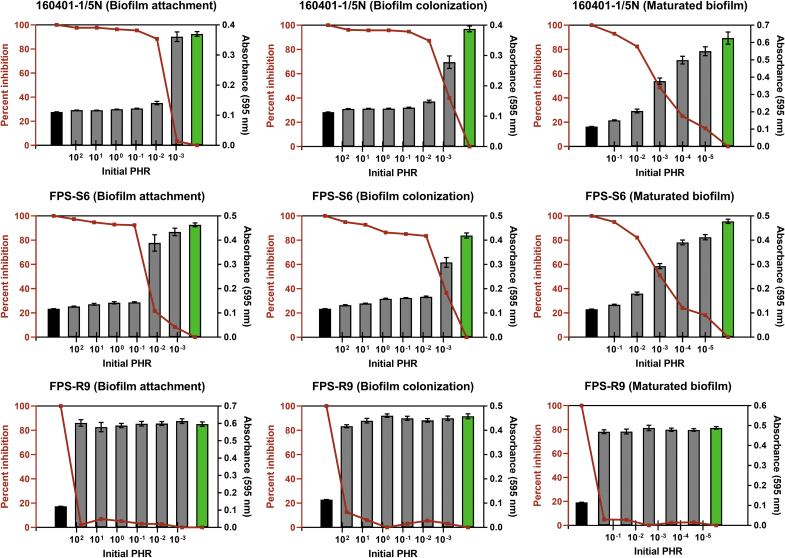
*Flavobacterium psychrophilum* biomass at different stages of biofilm formation measured by crystal violet staining (absorbance at 595 nm) after phage exposure at different initial PHRs. Each gray column represents the average (±SEM) biomass in six replicate wells on three replicate polystyrene plates. Green columns depict the biomass of the positive control (only bacteria, no phage exposure) and black columns the negative control (only phages, no bacteria). Red lines show the percentage inhibition of bacteriophage FPSV-D22 on three different stages (attachment, colonization, and maturation) of *F. psychrophilum* biofilm formation. Note that the experimental PHRs calculated from Tables 1 and 2 are relative to the exact position of the columns on the logarithmic *x*-axis. Isolate FPS-R9 was resistant to phage FPSV-D22 and, therefore, unaffected by the phage-exposure treatments. PHR, phage:host ratio; SEM, standard error of the mean.

The concentration of phage FPSV-D22 (Table 1) and *F. psychrophilum* (Table 2) was calculated before each phage-exposure treatment to estimate the minimal PHR required for efficient inhibition of *F. psychrophilum* attachment and colonization, and disruption of maturated biofilms (Fig. 1). Our experiments showed that an initial PHR of 0.1 and above almost completely inhibited (>80%) all stages of biofilm formation of the phage-susceptible isolates FPS-S6 and 160401-1/5N. Interestingly, in all three formation stages of these two isolates, except from the attachment phase of FPS-S6, an even lower PHR (0.01) was enough to inhibit biofilm formation by at least 80%. Owing to the high bacterial concentration of the maturated biofilms, the initial PHR was <1 even at the highest tested concentration of FPSV-D22. Still, a PHR of 0.1 and 0.01 almost completely eradicated the maturated biofilms of FPS-S6 and 160401-1/5N (Fig. 1). In most cases, an initial PHR of ≤0.001 was ineffective (<50%) in inhibiting *F. psychrophilum* biofilm formation. The phage-resistant isolate FPS-R9 was expectedly unaffected by each phage-exposure treatment.

### Efficacy of bacteriophages on survival of experimentally infected fish

**FIG. 2.**
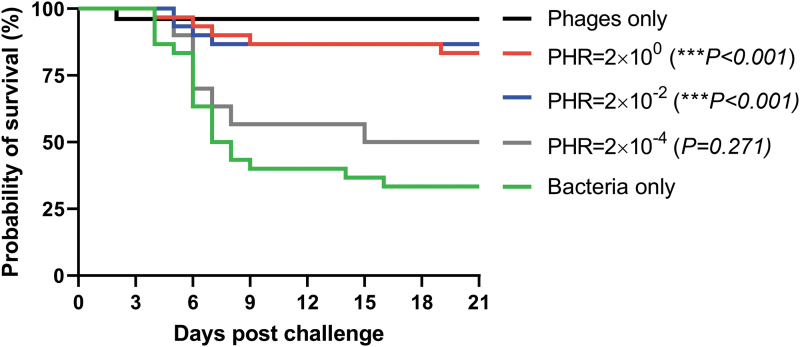
Kaplan–Meier survival curves for groups of rainbow trout treated with an i.p. injection of bacteriophages FPSV-D22 and FpV4 at a PHR of 2, 0.02, and 0.0002 one day after experimental i.p. infection challenge with *Flavobacterium psychrophilum* isolate 950106-1/1. The survival curves of each treatment group were compared with the mock-infected control group using the log-rank (Mantel–Cox) test followed by pairwise comparison using the Gehan–Breslow–Wilcoxon test. Stars indicate significant (*p* < 0.001) differences in the probability of survival between the treatment groups (PHR = 2 × 10^−2^ and PHR = 2 × 10^0^) and the control group (bacteria only) at the 99.9% level. i.p., intraperitoneal.

After the experimental infection, the first mortalities were recorded 4 days postchallenge with *F. psychrophilum* and the diseased fish that were examined showed typical signs of BCWD with splenomegaly and tissue necrosis. At the end of the challenge experiment, the mean cumulative percentage (± standard deviation) mortality in the group receiving only bacteria was higher (67% ± 5.8) compared with the treatment groups injected with bacteriophages at a PHR of 2 (17% ± 11.5), 0.02 (13% ± 5.8), and 0.0002 (50% ± 0). The RPS after treatment with bacteriophages in a PHR of 2, 0.02, and 0.0002 was 76%, 81%, and 26%, respectively. The probability of survival (%) (Fig. 2) in the group receiving only bacteria (33%) was significantly lower (*p* < 0.001) compared with the treatment groups injected with phages and bacteria at a PHR of 2 (83%) and 0.02 (87%). The probability of survival in the group treated with bacteriophages in a PHR of 0.0002 did not differ statistically (*p* = 0.271) from the control group. One fish injected with only phages died early in the experiment due to injury during injection. Positive identification of *F. psychrophilum* isolated from internal organs of the experimentally infected fish was verified by PCR using species-specific primers.

## Discussion

In recent years, there has been a growing interest in application for phage therapy in aquaculture, but only a few phage products have so far been made commercially available.^15^ In rainbow trout production, *F. psychrophilum* is mainly a problem for newly hatched fry, which are immunodeficient and unable to be vaccinated, and which are subjected to recurring infections that require the use of medicated feed. Similar to many opportunistic pathogens in aquaculture, *F. psychrophilum* remains often undetected until fish become susceptible to infection. Exactly what causes or triggers outbreaks of BCWD in fish farms is not known, but disease outbreaks are known to occur shortly after stress associated with handling and transport of fish. There is also strong evidence to support that virulent *F. psychrophilum* strains are spread geographically with the international trade of live fish eggs.^16,17^ Recurring outbreaks of BCWD in aquaculture settings may then occur through contaminated rearing water or tank surfaces, or by horizontal transmission from egg to egg or fish to fish. Good management and husbandry practices, such as egg and tank surface disinfection procedures, particularly during fry rearing, may show beneficial effects but are usually not enough to control disease outbreaks. The eradication of *F. psychrophilum* from contaminated eggs or from surfaces where it may reside in biofilms is hampered by the intrinsic resistance of the pathogen to the most commonly used disinfectants (iodophors).^18,19^

The efficacy of phage therapy partly relies on the capability of phages to connect with their hosts, which is not necessarily occurring in open aquaculture systems. However, administration of phages in closed recirculated aquaculture systems (RAS) or more locally on contaminated surfaces, or in hatchery trays with restricted dilution capacity increases the chance of encounter between phage and the target bacterium. It was recently shown that *F. psychrophilum* can form biofilms on different types of materials used on rainbow trout farms, including stainless steel, plastic, glass, and wood.^2^ Our results showed that phage FPSV-D22 was highly efficient in preventing and interrupting *F. psychrophilum* biofilm formation, and in disrupting maturated biofilms on inert plastic surfaces even at low phage:bacteria ratios (≥0.01) when applied locally *in vitro* in the absence of natural dilution processes. Before FPSV-D22 or similar phages are suitable for treatment of live eggs or fry, which are particularly vulnerable to *F. psychrophilum*, the safety of the phage preparation needs to be ensured as it is known that crude phage lysates may contain bacterial endotoxins.^1,20^ However, low toxicity of the purified phage suspension used in our phage therapy trial is expected due to the normal swimming and feeding behavior exhibited by fish that received a 100 μL injection of a high dose (1.7 × 10^8^ PFU/fish) of phages only and the single early mortality event in the group, which was presumably due to misinjection.

Our study also shows that injection of lytic bacteriophages can significantly reduce mortality caused by *F. psychrophilum* in experimentally infected juvenile rainbow trout and that the level of protection is dose dependent. The therapeutic potential of phages FpV4 and FPSV-D22 was further emphasized by the fact that their lytic potential against isolate 950106-1/1 were relatively low (>1000-fold less efficient), compared with their infectivity against the FPS-S6 and 160401-1/5N isolates used in the biofilm experiments.^8^ Previous studies have indicated that an initial PHR of at least 10 is required for rapid and effective control of *F. psychrophilum* infections in fish.^7^ However, in liquid cultures, such high PHRs select for phage resistance in the bacterial host population, whereas phage-sensitive clones dominate (>99.8%) the regrowing population at lower (≤0.5) PHRs.^21^ Interestingly, in our study a PHR of 2 and 0.02 elicited equal therapeutic effects in fish, which significantly (*p* < 0.001) increased the probability of survival in treated fish, indicative of *in vivo* multiplication of the phages. Together, these results indicated that a high initial phage encounter rate is essential for the efficiency of the phage control. We suggest that even at the low initial PHR in the current experiment, the restricted dispersal of phages and bacteria and a high local encounter rate in the biofilm and the i.p. infected fish, compared with the dilute liquid environments previously tested, ensured an efficient phage infection of the host.^21^ The high RPS values 76% and 81%, respectively, after treatment with bacteriophages in a PHR of 2 and 0.02 indicate that with an effective delivery method, phages could also be used for prevention or treatment of BCWD outbreaks in fish farms. Therefore, the next step would be to determine whether a protective effect of phages can be obtained using a more practical delivery route, for example, through feed or immersion. A phage therapy trial with *F. columnare* showed that a single addition of a specific phage preparation into the water in a flow-through fish tank system increased the percentage survival of rainbow trout significantly in the phage treated group (50%) compared with the nontreated control group (8.3%).^22^ These findings indicate that phages have a strong potential for use against infections caused by *Flavobacterium* species, particularly as phages targeting these species have shown to be able to persist in increasingly popular closed RAS.^23^

Studies mimicking phage therapy of natural BCWD outbreaks are at least in part hampered by the lack of a reproducible challenge model that mimics natural disease and does not involve injection of *F. psychrophilum* into fish. Also, several virulent *F. psychrophilum* strains resistant to several broad-range lytic phages, such as FPS-R9 used in this study, have been isolated from BCWD outbreaks.^8^ For control of *F. psychrophilum* in fish farms, the development of phage resistance is maybe an easier issue to overcome since phages are evolving with their hosts in the environment and thus new ones can be isolated, whereas phage-resistant *F. psychrophilum* clones show significant decrease in fitness and pathogenicity.^24^

## Conclusions

Our study shows that specific bacteriophages can elicit therapeutic effects against experimental *F. psychrophilum* infections *in vivo* and different stages of biofilm formation *in vitro* even at low PHRs (∼0.01). These findings can be used as a basis for further phage therapy testing through the use of different delivery routes.
